# Three-dimensional mouse brain cytoarchitecture revealed by laboratory-based x-ray phase-contrast tomography

**DOI:** 10.1038/srep42847

**Published:** 2017-02-27

**Authors:** Mareike Töpperwien, Martin Krenkel, Daniel Vincenz, Franziska Stöber, Anja M. Oelschlegel, Jürgen Goldschmidt, Tim Salditt

**Affiliations:** 1 Institute for X-Ray Physics, University of Göttingen, Göttingen, Germany; 2 Center for Nanoscopy and Molecular Physiology of the Brain, Göttingen, Germany; 3 Leibniz-Institute for Neurobiology, Magdeburg, Germany

## Abstract

Studies of brain cytoarchitecture in mammals are routinely performed by serial sectioning of the specimen and staining of the sections. The procedure is labor-intensive and the 3D architecture can only be determined after aligning individual 2D sections, leading to a reconstructed volume with non-isotropic resolution. Propagation-based x-ray phase-contrast tomography offers a unique potential for high-resolution 3D imaging of intact biological specimen due to the high penetration depth and potential resolution. We here show that even compact laboratory CT at an optimized liquid-metal jet microfocus source combined with suitable phase-retrieval algorithms and a novel tissue preparation can provide cellular and subcellular resolution in millimeter sized samples of mouse brain. We removed water and lipids from entire mouse brains and measured the remaining dry tissue matrix in air, lowering absorption but increasing phase contrast. We present single-cell resolution images of mouse brain cytoarchitecture and show that axons can be revealed in myelinated fiber bundles. In contrast to optical 3D techniques our approach does neither require staining of cells nor tissue clearing, procedures that are increasingly difficult to apply with increasing sample and brain sizes. The approach thus opens a novel route for high-resolution high-throughput studies of brain architecture in mammals.

The quantitative understanding of tissue functions requires three-dimensional (3D) structural information down to the (sub-) cellular scale. The structure and neuronal connectivity in the central nervous system or peripheral nervous system are excellent examples. Well established imaging techniques like histological sectioning^1^, light sheet fluorescence microscopy^2^, magnetic resonance imaging^3^, and more recently serial block-face electron microscopy^4^ provide important data, but require either invasive and extensive sample preparation or clearing techniques^5^ limiting throughput and volume or lack the required spatial resolution and contrast. A classical 3D imaging technique, predominantly used in daily clinical practice, is x-ray computed tomography (CT). Conventionally, contrast is based on absorption of x-ray radiation while passing through the sample. This is very well suited to visualize dense or mineralized tissue such as bones, but often yields insufficient contrast for soft tissue. For this reason, it becomes necessary to exploit the phase shift additionally to the absorption. Indeed, the real part of the x-ray index of refraction *n*(**r**) = 1 − *δ*(**r**) + *iβ*(**r**), the decrement *δ*, which gives rise to a sample-induced phase shift in the beam, is up to 1000 times larger for hard x-rays and soft tissue than the extinction coefficient *β*, which determines the attenuation coefficient. There are several full field imaging methods to record object induced phase distributions, such as Zernike based phase contrast^6^, Talbot interferometry^7^, edge-illumination^8^ or speckle-based phase contrast^9^, among others. Best suited for microscopic resolution, formation of phase contrast by free-space propagation behind the object has been used since third generation synchrotron radiation became available^10^,^11^,^12^. Recent progress in reconstruction algorithms and x-ray optics has brought this approach to a resolution range of 20–50 nm, for test objects and biological cells, respectively^13^. Importantly, the aforementioned imaging techniques are also compatible with the low partial coherence provided by laboratory microfocus sources^9^,^14^,^15^,^16^,^17^. Compared to interferometric techniques, propagation-based phase contrast offers higher resolution, conveniently in the range of a few micron to the sub-micron range, while simultaneously requiring a lower dose^18^,^19^. However, the quality of image contrast and reconstruction under the challenging setting of low coherence has remained in doubt^18^.

Here, we present propagation-based phase-contrast tomography at an optimized combination of a liquid-metal jet laboratory source^20^, instrumentation, sample preparation and reconstruction algorithm, to image large volumes of brain tissue from mice. We can show that high contrast within the reconstructed volume can be achieved without labeling and that within a region of interest (ROI) individual neurons can be visualized. This gives access to the intact 3D cytoarchitecture of the brain.

In our novel sample preparation technique we place the tissue in xylene after dehydration in ethanol, similar to conventional protocols for paraffin embedding. As a crucial and novel step we omit any further embedding or contrasting procedure and measure the specimen after evaporation of the organic solvent. In contrast to staining with high-Z elements, this technique, which we term EOS (evaporation-of-organic-solvent)-preparation, substantially lowers absorption due to removal of water and lipids while generating a novel contrast between the tissue protein-matrix and surrounding air.

Reconstruction starts with phase retrieval of projection images recorded for each angle *θ* of a tomographic scan. For objects with a slowly varying absorption at small propagation distances *z* behind the object, the intensity distribution can be expressed by an approximated form of the transport of intensity equation^21^


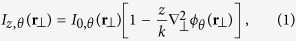


where *I*_0,*θ*_(**r**_⊥_) and *ϕ*_*θ*_(**r**_⊥_) denote the intensity and phase distribution directly behind the object and 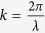 the wavenumber. Due to the laplacian, phase contrast typically appears in this regime as edge-enhancement. For weakly absorbing objects, the phase information can be retrieved by applying a 2D Fourier filter of the form 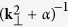 to the measured intensity images, with the spatial frequencies **k**_⊥_ and the absorption-dependent regularization parameter *α*. This reconstruction scheme, called the Modified Bronnikov Algorithm (MBA), provides good results for the given assumptions but leads to blurred reconstructions for non-negligible absorption^22^. However, the hereby retrieved approximated phase 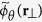 can be used to obtain a sharp reconstruction of the intensity distribution directly behind the object via


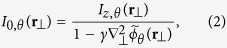


where 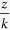 was replaced by the *α*-dependent regularization parameter *γ*. We found that this reconstruction approach, proposed by *Witte et al*. and known as the Bronnikov Aided Correction (BAC)^22^, gives superior results for the present unstained tissues and the used microfocus CT setup^23^. It is hence used as the default algorithm in the present work (Fig. 1d,e). After applying the phase-retrieval algorithm on each projection of the tomographic scan, 3D reconstruction is carried out with a (cone-beam) filtered back-projection^24^. Note that the contrast of the reconstructed object must be considered as an effective quantity, firstly since both absorption and phase interaction contribute to its values, and secondly since for flux reasons lab-CT typically utilizes broad bandpass radiation in contrast to synchrotron CT.

In a setup consisting of a point-like source the required high resolution of the imaging system can either be achieved via a high geometrical magnification *M (z*_1_ ≪ *z*_2_) and simultaneously smaller lateral source dimensions, allowing for measurements with selectable zoom and resolution^25^, or via a lower magnification (*z*_1_ ≫ *z*_2_) and smaller detector PSF^17^,^26^. Although the minimal source dimensions in modern nano- and microfocus sources are becoming impressively small, the limitations in flux for these small spot sizes lead to unfeasible exposure times for biological specimens. Liquid-metal jet sources on the other hand have a high flux but cannot provide the sufficient spot sizes to compete with the PSF of lens-coupled detector systems. Therefore we turn to the second approach, which we denote as the *inverse geometry* (Fig. 1a). In this mode the blurring due to a finite focal spot size, also known as the penumbra blur, is minimized as the sample-to-detector distance *z*_2_ is small compared to the source-to-sample distance *z*_1_. The resolution is in this case limited by the detector pixel size as well as vibrations in the setup and the highest resolution achieved with the imaging system presented here is about 800 nm in 2D, as qualitatively determined by a test pattern (Fig. 1b). Complementing this high resolution setting, the setup can also be employed in a lower resolution cone-beam geometry mode, allowing for overview scans with a larger field of view.

We demonstrate the capabilities of the laboratory setup by imaging different regions of interest in a wild-type mouse brain.

## Results

The first sample is the right hemisphere of a mouse brain which was cut out of the whole brain in order to get a smaller sample volume and therefore minimize absorption. For an overview, a tomogram in cone-beam geometry was recorded with a field of view large enough to cover the whole sample. A volume rendering of the reconstructed volume as well as a coronal and a horizontal slice are depicted in Fig. 2a–c. Prominent structures like the hippocampus are already visible but not resolved in cellular detail. To improve the resolution, regions of interest selected based on the overview scan, were imaged in inverse geometry. Note that in this case the imaged part of the sample was not cut out of the larger volume but that a true region-of-interest tomography scan was performed. In this case typical artefacts at the edges of the slices may arise but can be prevented by continuously extending the projections with constant values^27^. A region of interest tomogram with 1000 projections over 180° was recorded in the area of the hippocampus as well as the cortex with a pixel size of 0.47 *μ*m and an exposure time of 50 s per projection. Two slices through the reconstructed volumes are shown in (d) and (f), revealing structural details at single cell resolution in a quality comparable to classical histology results. In contrast to histology, this resolution is not only available in thin slices cut from the sample but isotropically in the entire 3D volume. This allows for virtual slicing of the sample in every arbitrary orientation without affecting the 3D structure. Therefore it is also possible, in contrast to classical histology, to virtually produce sections of the same sample from different directions and gain a more complete image of the cytoarchitecture. The slice thickness is determined by the voxel size of the reconstructed volume, which in this case is 0.95 *μ*m. To emulate histological contrast in a 30 *μ*m thick section, a maximum intensity projection over 31 successive slices was generated for both high resolution datasets (Fig. 2e,g). Although already visible in the thin virtual sections, prominent features like the pyramidal cell layer and dentate gyrus in the hippocampus as well as the barrel field in the cortex are clearly distinguished at high contrast. Complementing the evaluation of the cyto-architecture, minimum intensity projections can be used to visualize the vascular architecture within the sections (Supplementary Fig. S1).

To further evaluate the imaging capabilities of the laboratory setup, the olfactory bulb, brain stem (Supplementary Figs S2 and S3) and cerebellar vermis of a mouse brain have been scanned as other prominent examples of the central nervous system. Figure 3a shows a transverse slice through the reconstructed volume of the central part of the vermis. The different layers, the white matter, the granular layer, the molecular layer and the purkinje cell layer, are clearly resolved in cellular detail, again showing a similar resolution and contrast as classical histology. The longitudinal slice shown in (b) also reveals to some extent axon bundles within the white matter. For a better understanding of the 3D architecture of the sample an automatic volume rendering of the sample is shown in Fig. 3c, revealing the typical folded structure of the cerebellum. As the resolution allows for the segmentation of single cells, even the cellular distribution within the different layers can be imaged in 3D, see Fig. 3d. Since the segmentation was performed manually at this stage, only a small part of the sample was considered (Fig. 3c and Supplementary Video 1). However, at the demonstrated resolution, these cells could in principle be visualized for the entire sample.

As a benchmark and for comparison, we have also carried out measurements of a similar sample at the ID19 beamline at the ESRF in Grenoble (Supplementary Fig. S4, Table S1 and Video 2). The comparison of the reconstructed volumes suggests that, despite the lower brilliance of the laboratory setup as well as the long exposure times, they are of comparable quality and resolution, both showing cellular details in mm sized volumes.

## Discussion

The results demonstrate that the combination of a new tissue preparation and optimized propagation-based phase-contrast tomography enables imaging of the central nervous system at large scales with contrast and resolution high enough to identify single neurons, both at synchrotrons as well as laboratory sources. In principle, the novel tissue preparation, in its first steps, closely follows simple and inexpensive traditional ways of tissue embedding (as e.g. in paraffin embedding) indicating its wide applicability. Evaporation of the organic solvent as an additional step leaves a dried tissue matrix in which water and lipids have been removed. We here focus on the anatomical contrast generated with this method without further staining. As the brain preparation reduces absorption by an order of magnitude it can facilitate 3D X-ray fluorescence studies by reducing self-absorption. In fact, the rationale behind developing this technique was to facilitate X-ray fluorescence detection of thallium, which can be used as a marker of neuronal activity^28^. It is noteworthy, in addition, that the removal of lipids substantially promotes diffusion of compounds for counterstaining during immersion. Entire large brains could thus be stained after lipid removal with xylene (or other organic solvents) and subsequent rehydration. It is also of note that the first steps of dehydration and lipid removal mimic those in optical clearing techniques^2^. This could open a route for combining imaging of distinct labeled cell populations after optical clearing with subsequent CT-imaging of the cytoarchitecture.

This new form of virtual 3D histology avoids the drawbacks of classical histology, like labour-intensive and invasive sample preparation or mechanical distortions due to the slicing procedure, and provides virtual histological sections in any desired orientation and thickness. Most importantly, structure analysis can be carried out in 3D rather than 2D, allowing to address issues of neural connectivity as well as quantitative geometric analysis.

Classical histological staining of serial sections still forms the basis of analyzing brain structure in large numbers of studies dealing with phenotyping mutant mice or experimentally induced pathological alterations in rodent models of brain diseases. Compared to optical 3D techniques our approach offers advantages in view of high-throughput studies as it does not require staining or labeling of cells which are time-consuming steps in the sample preparation. Moreover, the limiting factor of relatively long scan times in our current protocol is likely to be overcome in the future as a good signal-to-noise ratio may already be achieved with shorter exposure times (see Supplementary Fig. S5) and several improvements of the liquid jet anode technology are expected to result in a significant increase in source brightness. Additionally, if beamtime access is available, the experiments can be carried out at the synchrotron, enabling a fast imaging of the samples at slightly higher contrast and resolution.

Note that the way we achieve high resolution and contrast suggests—with respect to both the tissue preparation and the underlying physics -, that protocols can be developed for studying 3D cyto- and fiber-architecture in the human brain. The low absorption of the preparation greatly facilitates measurements of large specimen.

The possibility to achieve the high resolution and contrast with laboratory sources makes our method applicable for a broad range of biomedical studies, requiring higher accessibility and throughput.

## Methods

### Sample preparation

**Figure 1:**
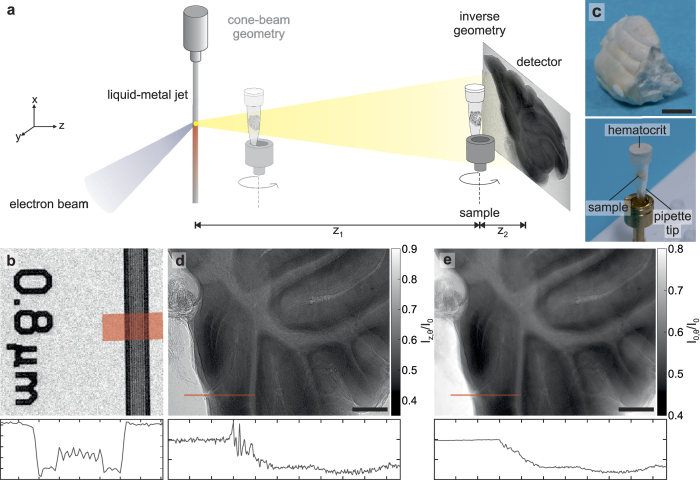
Laboratory setup **(a)** Schematic of the setup, in which X-rays are generated by a liquid-metal jet microfocus source. Depending on the ratio between the source-to-object and object-to-detector distances, it is either implemented in a cone-beam geometry or an inverse geometry imaging mode. **(b)** Image of an absorbing test pattern with 800 nm lines and spaces, together with the corresponding line profile along the structure (width: 50 px). **(c)** Close-up of a detached (mouse) cerebellar vermis and mounting of the sample in a pipette tip. **(d)** Typical empty-beam corrected projection of the sample in inverse geometry, with a line profile (width: 5 px) along an edge to illustrate the edge-enhancement effect. For a better signal-to-noise ratio, the projection has been resampled by a factor 2. **(e)** Reconstruction of the intensity distribution using the BAC algorithm. The line profile shows the advantage of the phase retrieval as the edge-enhancement is removed while simultaneously the signal-to-noise ratio increases. Scalebars: (**c**) 1 mm and (**d**,**e**) 250 *μ*m.

**Figure 2:**
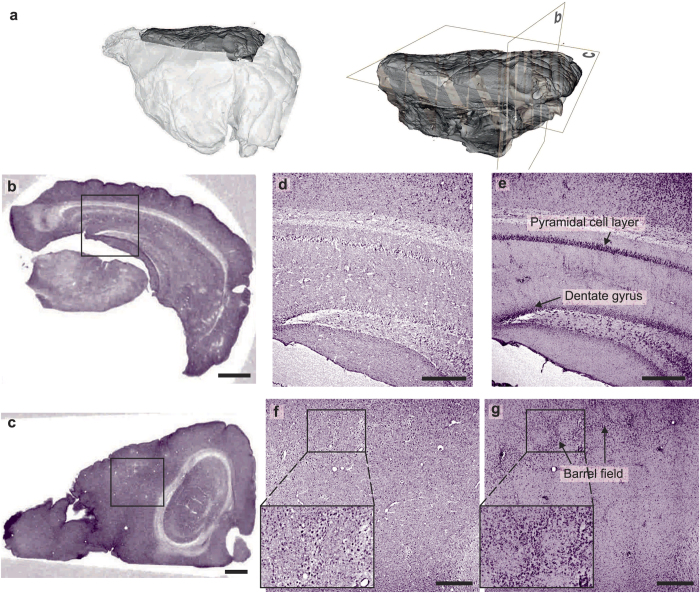
Combination of cone-beam and inverse geometry imaging modes **(a)** Volume rendering of the right hemisphere of a mouse brain, recorded in cone-beam geometry. The grey planes indicate the position of the slices shown in (**b**,**c**). **(b,c)** Coronal/Horizontal slice through the reconstructed volume. **(d)** Reconstructed coronal slice from the hippocampal region at cellular resolution, recorded in inverse geometry. The position of the high-resolution measurement in relation to the total sample volume is indicated in (**b**). Prior to the tomographic reconstruction the individual projections were resampled by a factor 2. **(e)** Maximum intensity projection of 31 successive slices, imitating a 30 *μ*m thick histological section. **(f)** Reconstructed horizontal slice from the cortical region. **(g)** Maximum intensity projection of 31 slices. Prominent cortical features as the barrel field are clearly visible. Scalebars: (**b**,**c**) 500 *μ*m, (**d–g**) 200 *μ*m.

**Figure 3:**
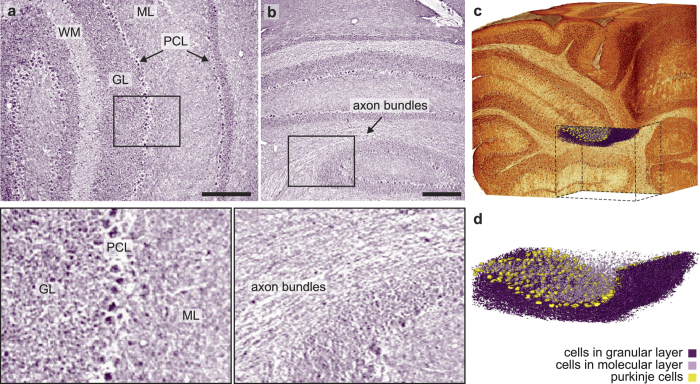
Volume rendering down to the cellular level of a mouse cerebellar vermis **(a)** Transverse slice through the reconstructed volume, showing the molecular layer (ML), granular layer (GL), white matter (WM) and Purkinje cell layer (PCL) of the cerebellar vermis at cellular resolution. Prior to the tomographic reconstruction the individual projections were resampled by a factor 2. **(b)** A longitudinal slice through the volume shows sufficient contrast to identify axon bundles within the white matter. **(c)** Automatic volume rendering of the sample with a cut within the volume, indicating the position of the cellular segmentation shown in (**d**). **(d)** Cellular segmentation of a small part of the sample. Scalebars: 200 *μ*m.

The brain preparation is based on dehydration of paraformaldehyde-fixed material in ethanol, removal of lipids in xylene and evaporation of xylene. We noted that the contrast was higher when dehydration was performed via transcardial perfusion with ethanol instead of conventional dehydration by immersion in ethanol. All experiments were in accordance with German ethical guidelines for the care and use of laboratory animals in experiments and approved by the local authorities (Landesverwaltungsamt Sachsen-Anhalt). Twenty adult mice were used for developing the paraformaldehyde/ethanol perfusion protocol. For brain preparation, adult mice were deeply anesthetized (induced with 2% Isoflurane, 0.75% O_2_, 0.55% N_2_O, followed by i.p. injection of 150 *μ*l of a Ketamin/Xylazin solution containing 12.5 mg Ketamin/ml and 0.5% Xylazin (Ketamin: Ratiopharm, Germany, Xylazin/Rompun: Bayer Healthcare, Germany)) and transcardially perfused with a short flush of saline (5 ml) followed by 15 min of phosphate buffered (100 mM, pH 7.4) 4% paraformaldehyde solution and 30 min with ethanol (≥99.8%, containing 1% methylethylketone, Carl Roth, Germany, termed ‘100% ethanol’ in the following according to common practice in histology). A peristaltic pump was used for perfusion. Flow rates were 15 ml/min during the first three minutes of perfusion (including perfusion with saline) and reduced to 7 ml/min during the remaining period. Animals were perfused with the different solutions without interrupting the flow. Brains were removed after perfusion and stored in 100% ethanol overnight in a refrigerator (7 °C). Brains were then transferred to a fresh solution of 100% ethanol and remained stored in this solution in the refrigerator for one to three days. Entire brains, or parts of the brains were then transferred to xylene (≥99%, p.a., isomers, for use in histology, Carl Roth, Germany). After overnight storage in xylene at room temperature the material was transferred to a fresh xylene solution and stored in this solution for one to two days at room temperature. The material was then placed on tissue paper under a hood at room temperature and dried overnight. For the experiments, the samples were carefully squeezed into a pipette tip to keep the sample position stable during the scan. In order to prevent sample changes due to external influences, a layer of hematocrit was deposited above the opening of the pipette tip, creating a closed chamber and therefore maintaining experimental conditions (Fig. 1c).

During development of the protocol brains not used for CT experiments were sometimes stored for several weeks in ethanol. We did not note any changes in drying behavior or gross morphology. Some of these brains were rehydrated (Supplementary Fig. S6). Rehydration times may depend on storage times in the different solutions and in the dried state. This was not systematically tested.

No changes in macroscopical appearance nor in contrast were observed when entire brains were dried as compared to smaller parts of the brain. Preparation of small-sized specimen suitable for mounting on object holders for CT was easier when samples were prepared before drying. We also noted that the entire procedure is applicable to large specimen (e.g. whole mouse bodies as the largest tested specimen). Evaporation of xylene resulted in tissue shrinkage but did not alter the gross morphology (Supplementary Fig. S6).

### Laboratory setup

A sketch of the generic laboratory setup is shown in Fig. 1. The liquid-metal jet source (Excillum, Stockholm, Sweden) consists of Galinstan (68.5% Ga, 21.5% In und 10% Sn) with the characteristic photon energy 9.25 keV (Ga-*K*_*α*_). For the experiments, it was operated at a tube voltage of 40 kV and the electrons were focussed to a size of 10 × 40 *μ*m^2^, resulting in an approximate focal spot size of 10 × 10 *μ*m^2^. The sample is positioned on a sample tower with three translational and one rotational axes for tomographic measurements. Additionally, two translations parallel and perpendicular to the beam allow for positioning of the rotation axis with respect to the optical axis. For alignment of the pitch angle and the zero position of the rotational axis, the detector is placed on a stage with two translations perpendicular to the optical axis.

In the inverse geometry the lens-coupled scintillator-based camera XSight (Rigaku, Tokyo, Japan) was introduced in the setup. It consists of a thin single-crystal scintillator, a 10-fold magnification objective and a 2504 × 3326 pixels CCD chip with a resulting pixel size of 0.54 *μ*m. For the experiments in cone-beam geometry a Flat Panel CMOS detector with a GdOS:Tb-scintillator screen (PerkinElmer, Waltham, USA) was installed. It consists of 1536 × 1944 pixels with a pixel size of 74.8 *μ*m. The geometric as well as experimental parameters for the measurements are listed in Supplementary Tab. S2.

### Data processing (laboratory setup)

Phase retrieval was performed with the BAC algorithm for all recorded projections using the parameters listed in Supplementary Table S2. Prior to the reconstruction of the 3D volume a wavelet-based ring removal algorithm was applied to the sinograms^29^. The 3D reconstruction of the volume was obtained with the UltraFast ConeBeam Reconstruction Software (Bronnikov Algorithms, Arnhem, The Netherlands). The visualization of the data was carried out with Avizo (FEI Visualization Sciences Group, Burlington, USA). The pink colorcode was chosen so that the virtual slices resemble histological sections stained with hematoxylin and eosin (H&E stain). The segmentation of the single purkinje cells was performed manually for a small part of the data set, whereas for the labeling of the cells within the molecular and granular layer a semi-automatical approach was used. Here, the cells were labeled with a threshold-based brush tool that, within the manually selected region by the user, only marks pixels with a gray value within a given range.

### Setup ID19 (ESRF)

The experiments were carried out at the ID19 beamline (ESRF, Grenoble, France) with a parallel beam geometry. The sample is positioned approximately 145 m behind the undulator on a sample tower with three translational and one rotational axes, while the position of the rotational axis can be adjusted via two additional translations. The energy of the setup is variable and for this experiment chosen as 18.685 keV with a flux of 
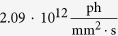
. The intensity distribution behind the sample is recorded with a lens-coupled scintillator based FReLoN (**F**ast **Re**adout **Lo**w **N**oise) CCD camera (ESRF, Grenoble, France) which is equipped with an optic revolver system (Optique Peter, Lentilly, France). The 10 *μ*m GGG:Eu scintillator converts the x-rays to optical light which reaches the CCD placed perpendicular to the optical axis via a mirror. It consists of 2048 × 2048 pixels with a size of 14 *μ*m which can be modified by the choice of objective directly behind the scintillator as well as eye-piece directly before the CCD, leading to a minimum pixel size of 280 nm. For the experiments shown here, we chose a pixel size of 0.7 *μ*m. To image the whole sample 12 individual tomograms at different positions on the sample were recorded and virtually stitched together. For stitching, the individual reconstructed subvolumes were loaded into the the software Avizo and overlapping parts between adjacent volumes were determined by looking for specific landmarks appearing in both datasets. After finding those landmarks the volumes were visually aligned and merged via the standard merging function in Avizo. The experimental parameters for a single tomogram are listed in Supplementary Table S2.

### Data processing (ID19)

As for the laboratory setup, phase retrieval was performed with the BAC algorithm for all recorded projections using the parameters listed in Supplementary Table S2. Prior to the 3D reconstruction a simple ring removal algorithm was applied^30^ which already delivered satisfying results and is significantly faster than the one used for the laboratory data. For the tomographic reconstruction the Matlab (Mathworks, Natick, USA) implemented function *iradon*, which performs a simple filtered backprojection, was used with the standard Ram-Lak filter. The analysis of the 3D data was again carried out with Avizo and the colorcode was chosen similar to the laboratory results. For the segmentation of the granular layer, the structure was labeled manually in approximately every third layer, followed by an interpolation by the software. Afterwards the labels were smoothed with a custom Matlab script and multiplied with the 3D data set, leaving only non-zero gray values within the granular layer. To get a 3D visualization of this data, a volume rendering was performed with a colormap, where regions outside of the granular layer are displayed as transparent. The cellular segmentation was performed in the same way as for the laboratory setup, again only for a small part of the sample (Supplementary Video 2).

## Additional Information

**How to cite this article**: Töpperwien, M. *et al*. Three-dimensional mouse brain cytoarchitecture revealed by laboratory-based x-ray phase-contrast tomography. *Sci. Rep.* **7**, 42847; doi: 10.1038/srep42847 (2017).

**Publisher's note:** Springer Nature remains neutral with regard to jurisdictional claims in published maps and institutional affiliations.

## Supplementary Material

Supplementary Information

Supplementary Video 1

Supplementary Video 2
