# Supplementary material for: Three-dimensional mouse brain cytoarchitecture revealed by laboratory-based x-ray phase-contrast tomography
Source: Sci Rep. 2017 Feb 27;7:42847. doi: 10.1038/srep42847 (PMC5327439; doi:10.1038/srep42847)
Supplement: Supplementary Information [file srep42847-s1.pdf]

Three-dimensional mouse brain cytoarchitecture revealed by laboratory-based x-ray phase-contrast tomography: Supplementary Information

Mareike Töpperwien[1,2], Martin Krenkel[1], Daniel Vincenz[3], Franziska Stöber[3], Anja M. Oelschlegel[3], Jürgen Goldschmidt[3,*], and Tim Salditt[1,2,*]

[1]Institute for X-Ray Physics, University of Göttingen, Göttingen, Germany
[2]Center for Nanoscopy and Molecular Physiology of the Brain, Göttingen, Germany
[3]Leibniz-Institute for Neurobiology, Magdeburg, Germany
[*]These authors contributed equally to this work. Correspondence regarding neurobiology should be addressed to J.G. (juergen.goldschmidt@lin-magdeburg.de), correspondence regarding x-ray imaging to T.S. (tsaldit@gwdg.de).

Vascular architecture

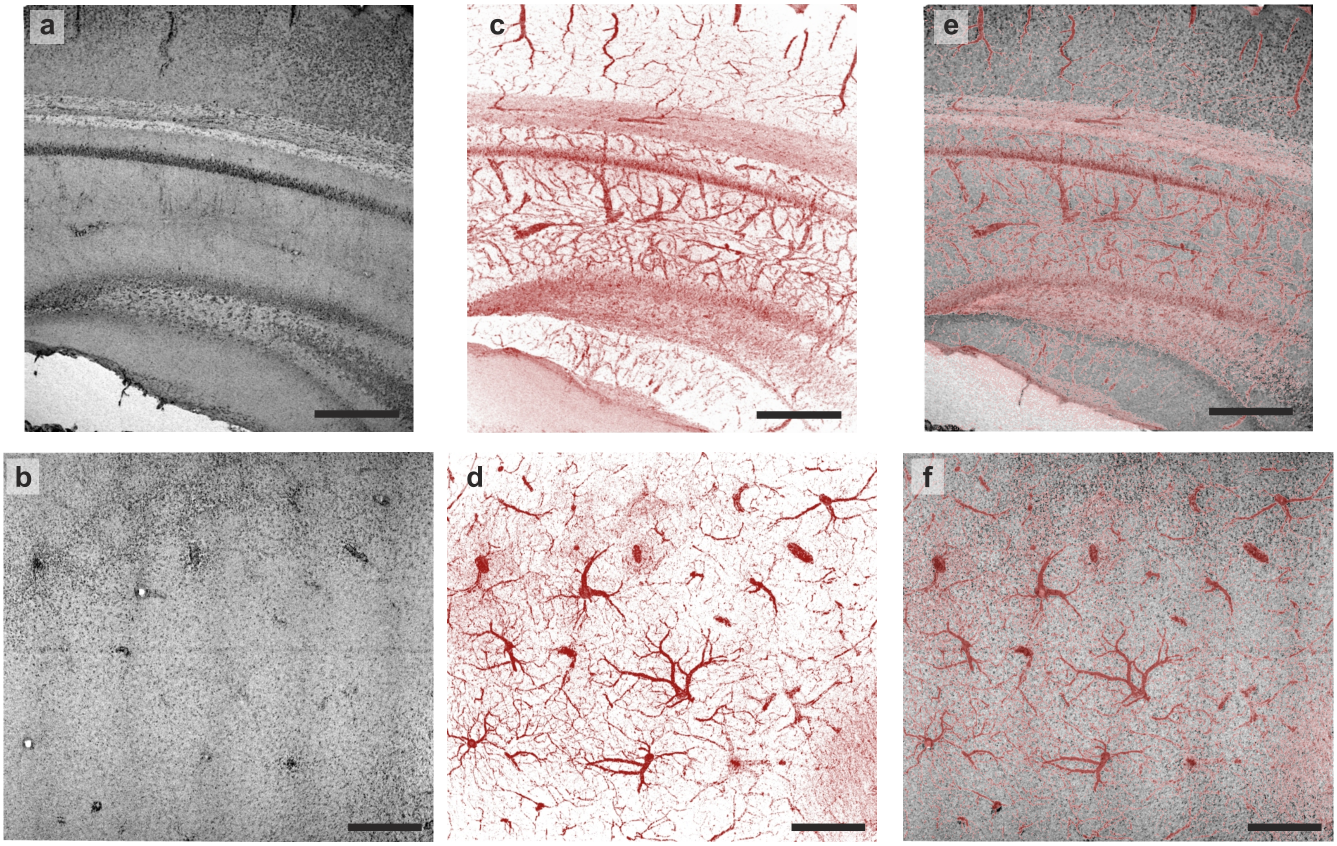

Figure S1. Visualization of the vascular architecture in thick section from the left hemisphere. **(a,b)** Maximum intensity projections over 85 successive slices in the hippocampal/cortical regions shown in Fig. 2 in the main text, simulating 80 μm thick histological sections. The locations of the slices are indicated in Fig. 2a-c **(c,d)** Minimum intensity projections over the same subvolumes. As blood is removed from the vessels during sample preparation, resulting in a smaller density compared to the surrounding tissue, the vascular architecture within the thick section is visualized. **(e,f)** Overlay of the vascular and cytoarchitecture of the sections in which white pixels are removed due to better visibility. Scalebars: 200 μm

Olfactory bulb

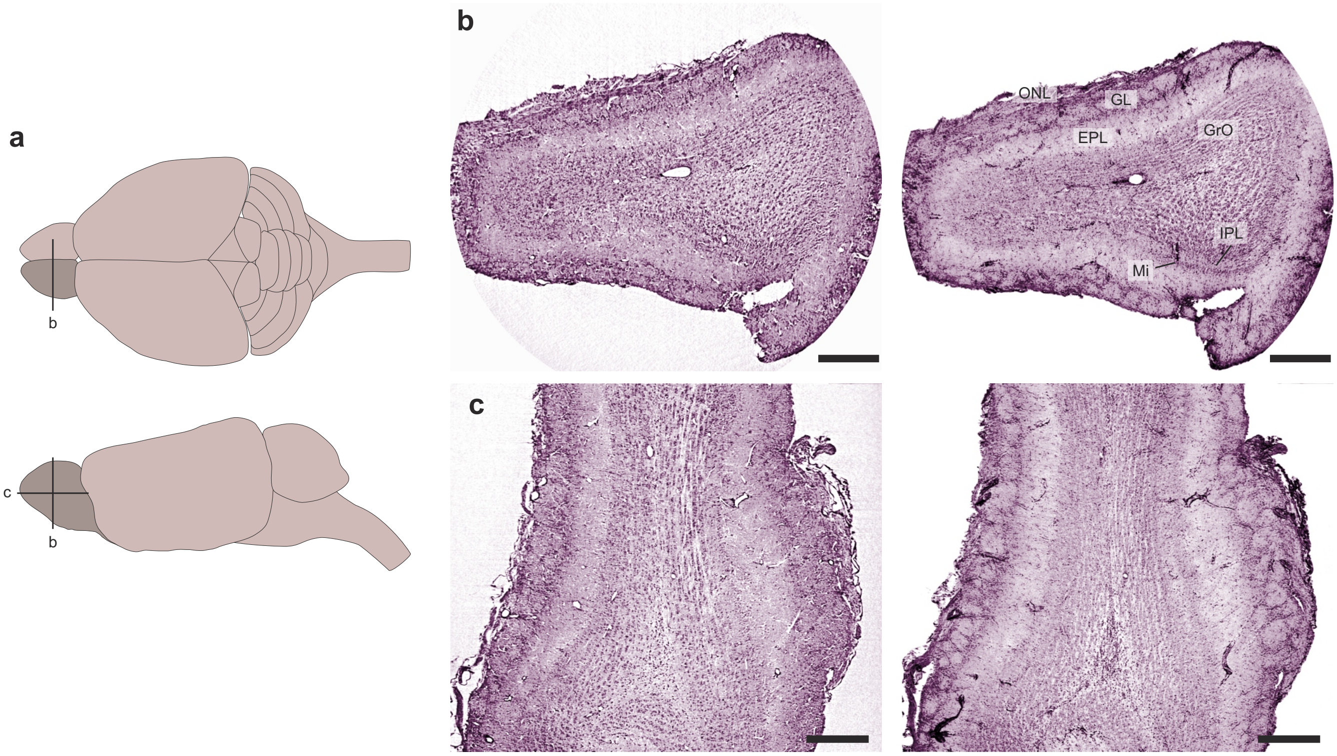

Figure S2. Imaging of the olfactory bulb (left half). (a) Sketch of a mouse brain, indicating the approximate positions of the sections shown in (b) and (c). (b) Coronal slice through the reconstructed olfactory bulb with an imitation of a 30 μm thick histological section by obtaining a maximum intensity projection over 31 successive slices. The typical layers of the olfactory bulb are well resolved at a resolution comparable to histology: Olfactory nerve layer ONL, Glomerular layer (GL), External plexifrom layer (EPL), granule cell layer of the OB (GrO), Inernal plexiform layer (IPL) and Mitral cell ayer (Mi). (c) Horizontal slice through the volume, revealing the same typical structures. Scalebars: 200 μm

Brain stem

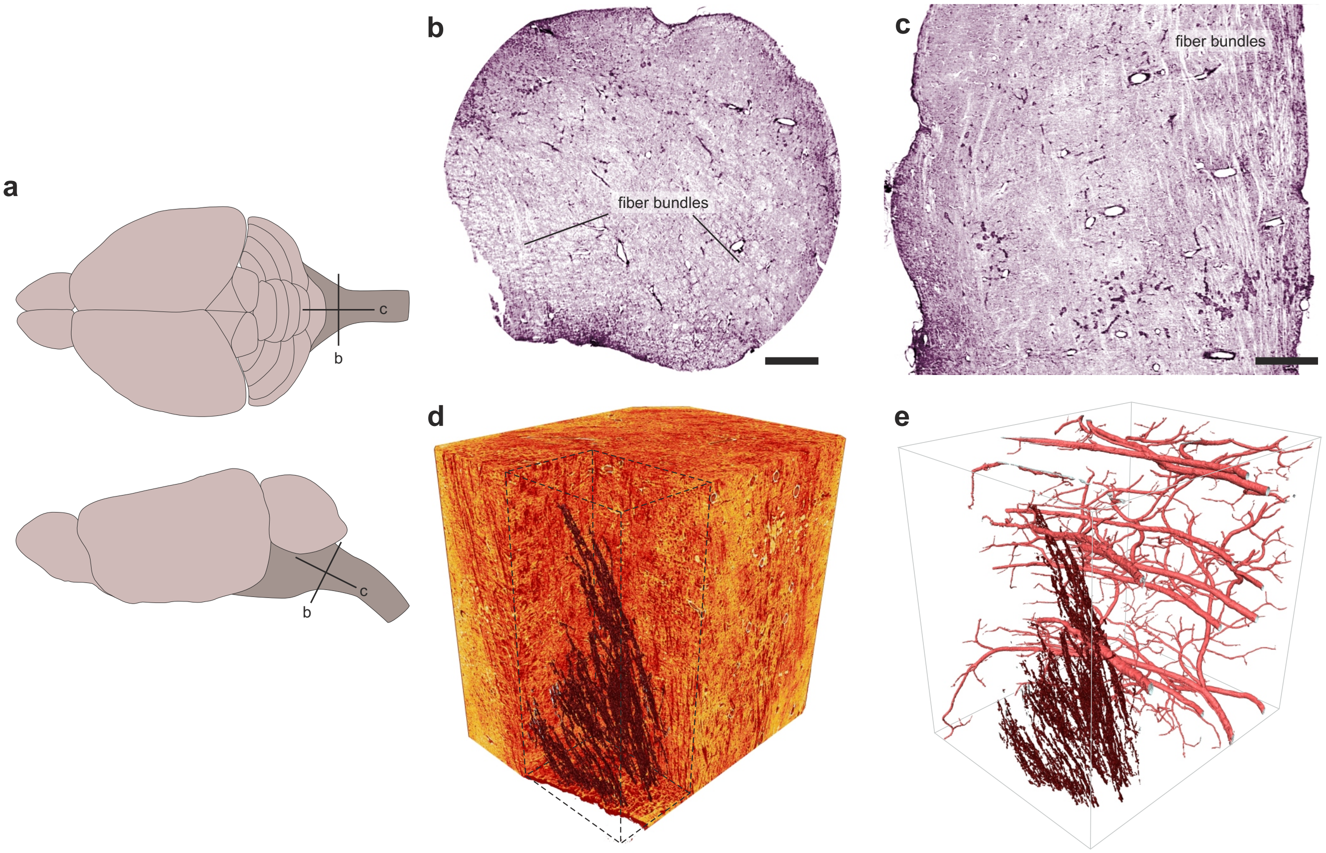

Figure S3. Imaging of the brain stem. (a) Sketch of a mouse brain, indicating the approximate positions of the sections shown in (b) and (c). (b,c) Coronal/sagittal slice through the reconstructed part of the brain stem, showing different features like the air-filled blood vessels or fiber bundles which can be recognized at the slightly lower electron density compared to the surrounding tissue. (d) Volume rendering of the reconstructed volume. Part of the fiber bundles are segmented automatically with a grey-value based segmentation tool and shown in dark red within the cut in the 3D rendering. (e) Additionally the vascular structure can be visualized in 3D as shown here for the largest blood vessels. Also the smaller vessels can be segmented automatically which is not shown here due to clarity of the figure. Scalebars: 200 μm

Synchrotron radiation phase-contrast CT

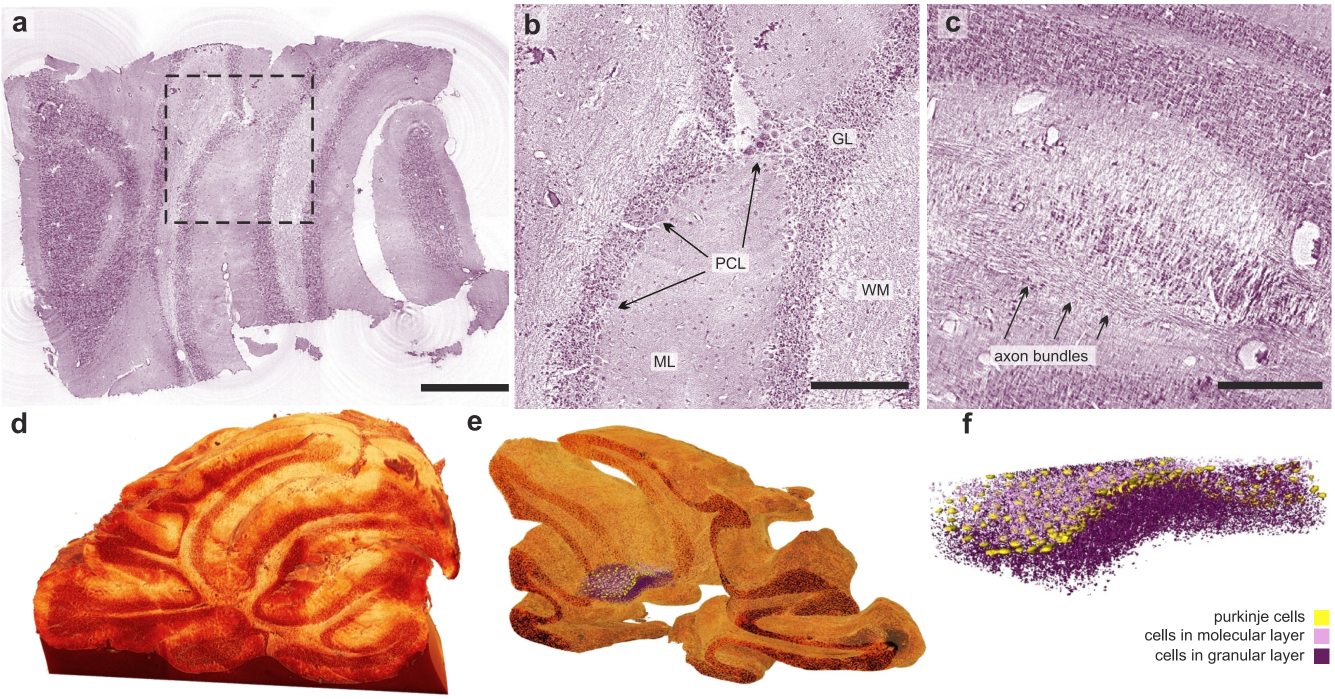

Figure S4. Results of the synchrotron radiation phase-contrast CT obtained at the ID19 beamline at ESRF. **(a)** Transverse virtual slice through the whole sample. For the reconstruction of the whole sample 12 individual tomograms were recorded at different positions on the sample and virtually stitched together. For a better signal-to-noise ratio the slices were resampled by a factor 2. **(b)** Close-up of the region indicated in (a). The white matter, granular layer, molecular layer and Purkinje cell layer are clearly visible at cellular resolution. **(c)** Longitudinal slice through the sample, showing axon bundles within the white matter. **(d)** Automatic grey-value based rendering of the whole virtually stitched volume . **(e)** Segmentation of the granular layer with density information included in the colormap, revealing the typical tight folding of the cerebellar tissue. **(f)** Single cell segmentation of a small part of the sample (as indicated in (e)). Scalebars: (a) 500 μm and (b,c) 200 μm

	total scan time	resolution [μm]	signal-to-noise ratio	surface dose [Gy]	availability
**Laboratory**	14 h	3.53	5.9	$2.6 \cdot 10^3$	always
**ID19**	2 min	1.75	8.9	$5.4 \cdot 10^4$	apply for beamtime

Table S1. Comparison between the laboratory and the ID19 measurement. Note that total scan time does not take overhead of the motors and detector into account. The half-period resolution was estimated via a Fourier Ring Correlation, a 2D version of the Fourier Shell Correlation[1], between the central slice of two independent 3D reconstructions, using the projections under even angles and odd angles, respectively. As visibility of the cells within the tissue is of importance, the signal-to-noise ratio was estimated by comparing parts of the molecular layer without cells, which were taken as background, and the cells, in this case of the granular layer. An estimate of the dose was performed following the approach in[2], assuming a homogeneous object consisting of the model protein $H_{50}C_{30}N_9O_{10}S_1$ with a density of 1.35 g/cm^3.

Effect of different scan times on data quality

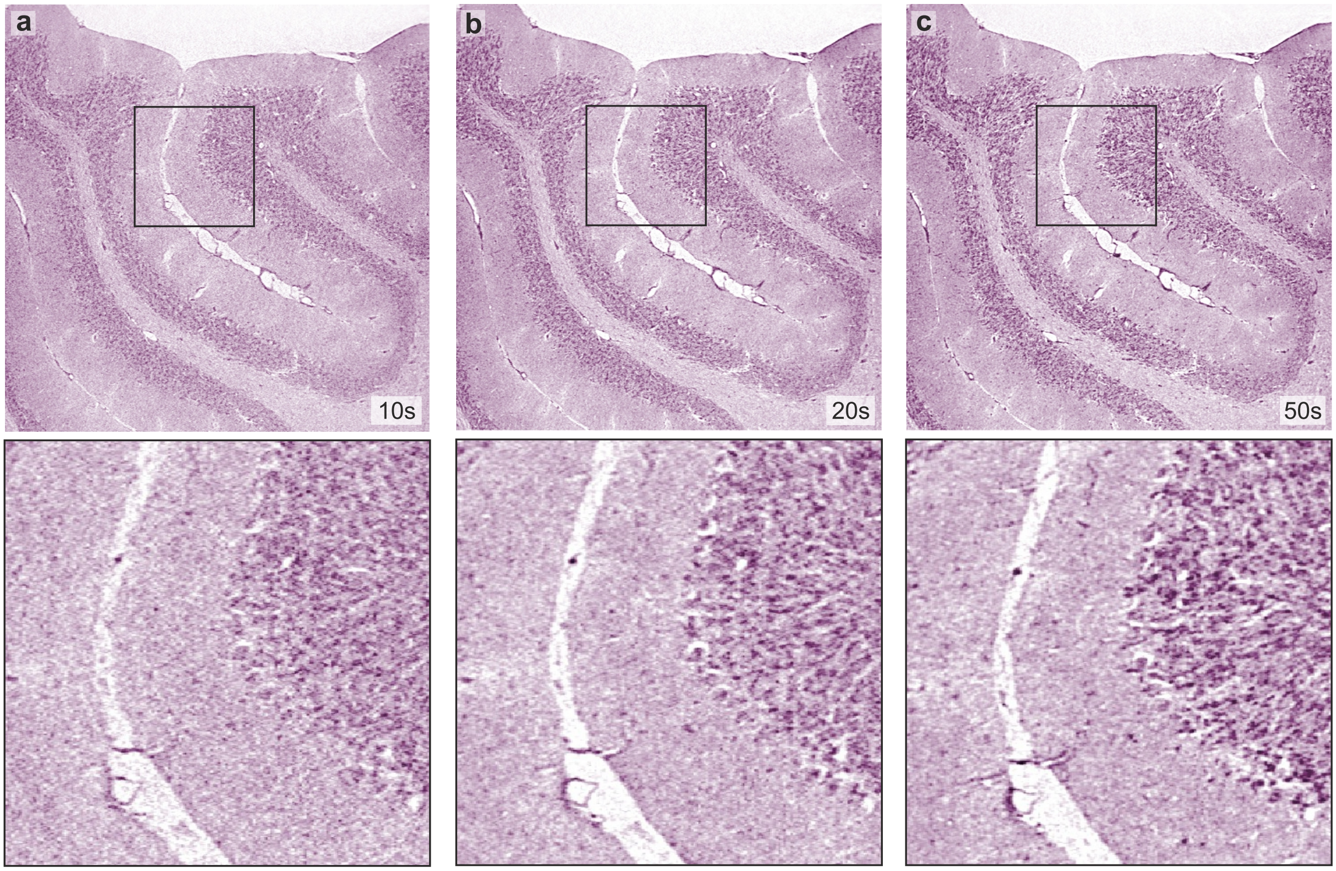

Figure S5. Comparison between datasets recorded with different exposure times. (a-c) Virtual slice as well as an inset at higher magnification through the volumes reconstructed from projections recorded with an exposure time of 10 s (a), 20 s(b) and 50 s (c). For all datasets approximately the same slice is shown. However, due to small changes between the measurements, the results are not entirely comparable. In order to get a better signal-to-noise ratio (SNR), all projections were resampled by a factor 2. The resulting SNR is estimated as in Tab. S1 and yields 4.1, 5.3 and 6.4, respectively.

Sample preparation

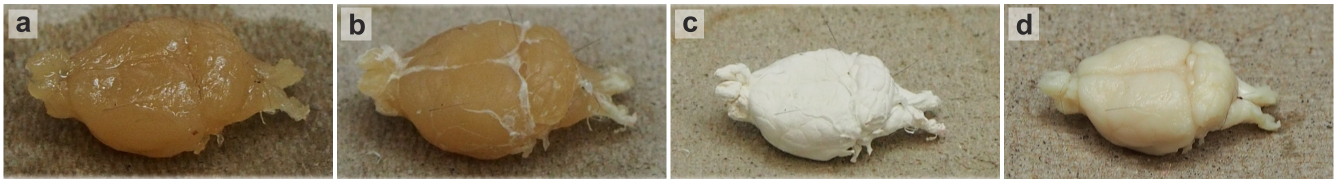

Figure S6. Four images of the same mouse brain at different steps in the sample preparation. (a) Directly after removing the brain from the xylene solution, (b) 12 min later, (c) 2 h after beginning of evaporation and (d) after three days storage in the dried state and one week rehydration in PBS.

Experimental parameters

**Figure**	**2(a-c)**	**2(d-f)**	**3**	**Supp. 2/3**	**Supp. 4**
Setup	Laboratory	Laboratory	Laboratory	Laboratory	ID19
Detector	Flat Panel	XSight	XSight	XSight	FReLoN
Pixel Size [μm]	74.8	0.54	0.54	0.54	0.7
z1 [mm]	100	158.75	158.75	158.75	
z2 [mm]	1767.3	22.6	18.3	22.6	20
M	186.73	1.14	1.12	1.14	
p_{eff} [μm]	4.02	0.47	0.48	0.47	
Field-of-view [mm^2]	6.17×7.8	1.18×1.57	1.2×1.6	1.18×1.57	1.4×1.4
Energy (spectrum)/K_α [keV]	0-70/9.25	0-40/9.25	0-40/9.25	0-40/9.25	18.685
e-Beam Power [W]	40	57	50	57	
Exposure Time [s]	1	50	50	50	0.1
Number of projections	916	1000	1000	1000	1000
Angular step	0.2	0.18	0.18	0.18	0.18
BAC, α	0.02	0.02	0.03	0.02/0.015	0.002
BAC, γ	0.13	0.65	0.475	0.56/0.62	0.4

Table S2. Experimental parameters.

Supplementary videos

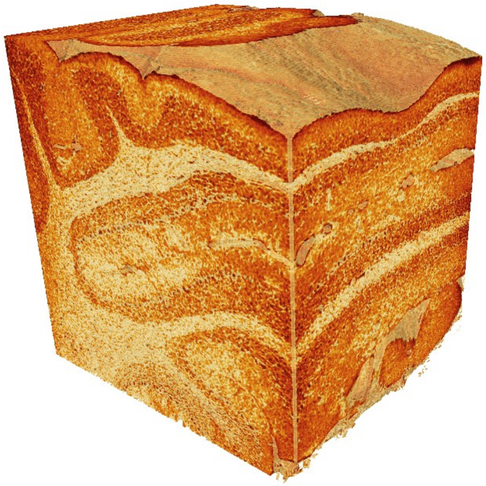

Video S1. 3D Visualization of the mouse cerebellum measured at the laboratory setup. In the beginning, the different transverse slices through the reconstructed volume are shown, followed by a 3D volume rendering of the entire volume and a cellular segmentation for a small part of the sample. In this cellular segmentation the cells in the granular layer are displayed in dark purple, the cells in the molecular layer in light purple and the purkinje cells in yellow.

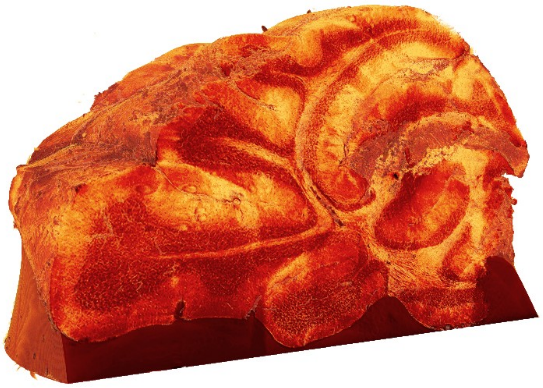

Video S2. 3D Visualization of the mouse cerebellum measured at ID19. In the beginning, the different transverse and longitudinal slices through the reconstructed volume are shown, followed by a 3D volume rendering of the entire volume and the granular layer. In the end, a cellular segmentation of a small part of the sample is displayed, where the cells in the granular layer are dark purple, the cells in the molecular layer light purple and the purkinje cells yellow.

References

1. van Heel, M. & Schatz, M. Fourier shell correlation threshold criteria. *J. Struct. Biol.* **151**, 250 – 262 (2005).

2. Howells, M. *et al.* An assessment of the resolution limitation due to radiation-damage in X-ray diffraction microscopy. *Journal of Electron Spectroscopy and Related Phenomena* **170**, 4–12 (2009).
